# Photochemical Properties and Stability of BODIPY Dyes

**DOI:** 10.3390/ijms22136735

**Published:** 2021-06-23

**Authors:** Patryk Rybczynski, Aleksander Smolarkiewicz-Wyczachowski, Jaroslaw Piskorz, Szymon Bocian, Marta Ziegler-Borowska, Dariusz Kędziera, Anna Kaczmarek-Kędziera

**Affiliations:** 1 Faculty of Chemistry, Nicolaus Copernicus University in Toruń, Gagarina 7, 87-100 Toruń, Poland; pat_ryb@doktorant.umk.pl (P.R.); 291065@stud.umk.pl (A.S.-W.); bocian@chem.umk.pl (S.B.); martaz@chem.umk.pl (M.Z.-B.); teodar@chem.umk.pl (D.K.); 2 Department of Inorganic and Analytical Chemistry, University of Medical Sciences, Grunwaldzka 6, 60-780 Poznań, Poland; piskorzj@ump.edu.pl

**Keywords:** BODIPY, photodegradation, DFT calculations, absorption spectrum

## Abstract

The present study is devoted to the combined experimental and theoretical description of the photophysical properties and photodegradation of the new boron-dipyrromethene (BODIPY) derivatives obtained recently for biomedical applications, such as bacteria photoinactivation (Piskorz et al., Dyes and Pigments 2020, 178, 108322). Absorption and emission spectra for a wide group of solvents of different properties for the analyzed BODIPY derivatives were investigated in order to verify their suitability for photopharmacological applications. Additionally, the photostability of the analyzed systems were thoroughly determined. The exposition to the UV light was found first to cause the decrease in the most intensive absorption band and the appearance of the hypsochromically shifted band of similar intensity. On the basis of the chromatographic and computational study, this effect was assigned to the detachment of the iodine atoms from the BODIPY core. After longer exposition to UV light, photodegradation occurred, leading to the disappearance of the intensive absorption bands and the emergence of small intensity signals in the strongly blue-shifted range of the spectrum. Since the most intensive bands in original dyes are ascribed to the molecular core bearing the BF_2_ moiety, this result can be attributed to the significant cleavage of the BF_2_ ring. In order to fully characterize the obtained molecules, the comprehensive computational chemistry study was performed. The influence of the intermolecular interactions for their absorption in solution was analyzed. The theoretical data entirely support the experimental outcomes.

## 1. Introduction

Photophysical and photochemical phenomena are nowadays indispensable and common in various fields of modern science, technology and medicine [1]. Among their applications of particular importance for everyday life comfort, the photocatalytic oxidation processes of environmental pollutants (for instance, bisphenol A, *p*-aminobenzoic acid, etc.) [2,3] or novel developments in photodynamic therapy (PDT) can be mentioned.

Photodynamic therapy has gained large interest, due to its weak side effects, as an alternative to damaging surgical, chemo- and radiotherapeutic treatment [4,5,6]. It is applied frequently in microbial illnesses, acne, psoriasis or several malignant types of tumors. PDT relies on the action of the photosensitive chemical agent, administered in darkness. When accumulated in the diseased tissues, it is irradiated with the light of a wavelength selected in order to prevent healthy tissue damage and at the same time, ensure the penetration of the infected tissues. It is commonly accepted that for safe and effective treatment, this absorbed wavelength should fit in the 700–1000 nm therapeutic window [4,5]. Under the influence of this light, the photosensitizer undergoes electronic excitation to the singlet excited state. Next, via the intersystem crossing, the triplet state of the photosensitizer can be generated and, further on its energy, can be transferred to the oxygen present in the cells to produce the reactive oxygen species (ROS) [4]. They are, indeed, the required potent agents for destroying diseased tissues.

The commonly applied PDT photosensitizers include porphyrins, chlorins and phthalocyanines, among others; however, their side effects, such as low bioavailability, susceptibility for aggregation, which satisfies the desired photophysical properties, or decomposition under light or oxygen presence induce further needs for improvements [4,5]. An effective photosensitizer is required to exhibit proper photophysical features, such as the following: strong absorption within the therapeutic window, preferably fluorescence together with negligible photobleaching; large intersystem crossing allowing for the high ROS generation quantum yield; the energy of the triplet state higher than the oxygen ground-state-to-singlet energy gap (>94.2 kJ/mol); and low dark toxicity [7]. Moreover, it needs to strongly accumulate in the diseased tissues, be soluble in water or allow for other drug transport media applications, ensure fast removal after therapy, and be synthesized in a stable form in an easy and reproducible synthetic pathway, which also implies its photostability during storage and therapy [4].

Presently, there is a wide variety of fluorescent and luminescent materials available, such as organic compounds, molecular complexes, biomacromolecules (such as proteins), and nanoparticles [8]. An example of such a fluorophore that has gained wide attention is 4,4-difluoro-4-bora-3a,4a-diaza-s-indacene (BODIPY). The extraordinary properties of this relatively simple system involve its extremely strong fluorescence, sharp emission and absorption bands and stability with respect to various environmental conditions (such as pH or solvent polarity), together with the easy tuning of its emission and absorption ranges by chemical modification with functional groups of the desired character. Since the first synthesis of this compound in 1968 [9], the BODIPY structure has been modified by many different functional groups [10,11,12]. The substituted compounds have found numerous potential applications, for instance, as a new agent in medical imagining [13,14] or as a photosensitizer for photodynamic therapy (PDT) [15,16]. Nevertheless, BODIPY dyes have so far been rarely applied in PDT, due to their weak absorption above 600 nm, poor water-solubility and photostability [17] as well as relatively inefficient intersystem crossing in the basic unsubstituted form [18,19]. However, due to their small internal conversion rate constant, extremely intensive fluorescence and easy chemical modifications that affect strongly their solubility and photophysical properties, they account for an attractive alternative as a PDT photosensitizer. Simple structural modifications of BODIPY dyes, such as substitution with heavy atoms, significantly increases the quantum yield of triplet state formation via spin orbit coupling. Additionally, the absorption and emission range of BODIPY can be rationally modified by proper substitution at 2, 6 and other positions in the molecular core. Only recently, BODIPYs were successfully applied for bacteria photoinactivation [20,21,22] and as photosensitizers in PDT, modified with a heavy atom (iodine, bromine, metal cation) for efficient triplet state population [4,7,23,24,25,26,27,28,29]. All of these works appeared in recent years, thus pointing out the possible practical applications of BODIPY in therapy and the need for their advanced developments.

**Figure 1:**
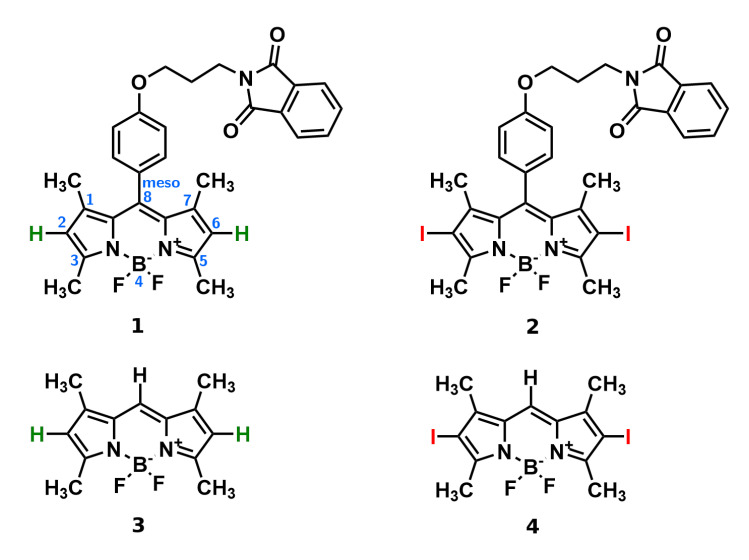
Structures of the **1** and **2** (iodinated) BODIPY dyes (atom numbering in the core presented in light blue); additionally structures of **3** and **4** prototypes presented below.

One of the important features, which need to be exhibited by efficient photosensitizers, is high photostability under UV and Vis radiation, together with easy photoactivation within the tissue. Therefore, it is crucial to determine the behavior of the photosensitizer under light, together with its careful absorption and emission characteristics—the determination of the photodegradation products is a required step in the conventional drug pre-formulation process. Despite the big interest in highly-fluorescent photostable BODIPY derivatives for biomedical applications, still only a few studies on the mechanisms of their photodegradation are available [30]. Most of them concentrate on the photodegradation kinetics, not considering the mechanism itself. However, this could be of vital importance for possible application in the photoactivated CO-releasing or NO-releasing BODIPY dyes or in the design of the photoactivation of dormant BODIPY photosensitizers directly in malignant cancer cells in order to avoid side effects [31,32,33,34]. The mechanism of BODIPY photodegradation involving the interaction of the dye in the triplet state with the generated singlet oxygen with production of the peroxo compound was described by Mula et al. [30]. Since oxidation is assumed to take place at C8 (compare Figure 1), sterical crowding in *meso-*position together with the presence of singlet oxygen quenchers has been shown to diminish the photodegradation rate.

According to our knowledge, the photodegradation phenomena of BODIPY dyes has been scarcely investigated so far with computational chemistry tools [19,30]. In most cases, the DFT approach has served as the way of estimation of the properties of the BODIPY dyes in their electronic ground state (such as charge distribution, orbital energies or dipole moment) or vertical absorption energy for the substrates. This arises from the complicated photodegradation mechanisms that occur frequently with the involvement of free radical species, which are highly challenging computationally and open numerous possible pathways for photodegradation reactions, including radical recombination, electron transfer processes, etc. Since BODIPY are known to be demanding with respect to the applied theoretical methodology, they require either advanced multiconfiguration treatment or the acceptance of only qualitative agreement of the corresponding TD-DFT results [19,35,36,37,38,39,40].

The two systems investigated in the present study were synthesized previously for possible biomedical applications for bacteria inactivation [22]. They differ in the substituents in the 2 and 6 positions—system **1** is unsubstituted, while system **2** contains iodine heavy atoms, incorporated in the structure to increase the intersystem crossing to the triplet state. This substitution is expected to quench the fluorescence quantum yield of the system and simultaneously increase the generation of singlet oxygen reactive species. Due to the synergistic action of both the BODIPY core, exhibiting green light absorption [41,42,43], and the phthalimide substituent of the known antimicrobial and anticancer properties, these systems are also expected to be attractive photodynamic therapy photosensitizers. As such, they should possess appropriate photostabilities and controllable degradation pathways under irradiation with visible light. Therefore, the aim of the present study is to determine the origin of the photophysical properties of the two antibacterial BODIPY dyes reported earlier [22] with the support of computational chemistry tools, and establish the connection between the possible photodegradation pathways and the observed modification of the absorption and emission spectra. Additionally, the influence of the solvent is investigated for broad solvent selection with different characteristics in order to ensure the stability of the dye in various environments.

## 2. Materials and Methods

### 2.1. Materials

Two investigated BODIPY systems are presented in Figure 1. Their synthesis follows the method reported earlier in the literature [22]. Compounds **1** and **2** were purified, using flash chromatography. It was carried out using a chromatographic system with a mass detector. Furthermore, the identity of the obtained products was confirmed, using NMR, UV–Vis spectroscopy and mass spectrometry. Organic solvents of high purity were obtained from Sigma-Aldrich and Avantor Performance Materials, Poland.

### 2.2. Methods

#### 2.2.1. Spectral Properties

The UV–Vis spectra were recorded in a quartz cell of 1 cm path length, using a Shimadzu UV160A spectrophotometer. The absorption spectra of the studied BODIPY dyes were recorded in the range from 200 to 1000 nm at room temperature. The emission spectra were obtained on a Jasco 6200 spectrometer. The diluted solutions of BODIPY **1** and **2** and a reference (absorbance at $\lambda_{max}$ about 0.2) were excited at 470 nm. A fluorescein solution in 0.1 M NaOH ($\Phi_{F,ref}$ = 0.92) was used as a reference for BODIPY [44]. The fluorescence quantum yields $\Phi_{F}$ were calculated according to the Equation (1), where F stands for the area under the emission curve, A is the absorbance at the excitation wavelength, and η corresponds to the refractive index, with the subscripts denoting the sample and the reference values [45]:

(1) $$\Phi_{F}=\Phi_{F,ref.}\cdot \frac{F_{sample}}{F_{ref.}}\cdot \frac{A_{ref}}{A_{sample}}\cdot \frac{\eta_{sample}^{2}}{\eta_{ref.}^{2}}.$$

#### 2.2.2. Photostability Studies

Photochemical stability under the UV–Vis irradiation of **1** and **2** was investigated at an ambient temperature, using MeOH as the solvent, in accordance with the previously described procedure [46]. A high-pressure mercury vapor lamp HPK 125 W (Philips, Amsterdam, The Netherlands), emitting polychromatic radiation in the range of 248–578 nm was applied with the incident radiation intensity 72 W/m^2^, 55 W/m^2^ and 18.7 W/m^2^ for UVA, UVB and UVC, respectively. Radiation intensity was measured with a radiometer HD 9021 (Delta, Italy) at the sample position, i.e., at 10 cm distance from the radiation source. The products of the photochemical reaction were identified, using the Shimadzu Prominence HPLC system (Tokyo, Japan). The instrument includes a binary solvent delivery system (LC-20 AD), an autosampler (SIL-20A), and a column thermostat (CTO-10AS VP), absorbance spectrophotometric diode array UV–Vis detector (SPD-M20A) and a data acquisition station. Separation was performed in gradient elution from 70% to 100% of methanol in water over 10 min on the Kinetex Phenyl-Hexyl (Phenomenex, Torrance, CA, USA) 2.1 × 100 mm chromatographic column with particle size 1.7 μm. The flow rate of the mobile phase was 0.25 mL/min, the injection volume was 10 μL, and the separation temperature was 35 °C. The investigated sample of **1** was separated with the HP-LC method, and for the photodegradation products, the UV spectrum was simultaneously registered. During the investigation, samples were collected after every 15 min of irradiation, starting with 5 min, then 20 min, and so on. The composition of the irradiated mixture was analyzed with the use of a DAD detector recording the change in the absorption of products leaving the chromatographic column. The wavelength range selected for the detector was 200–600 nm.

### 2.3. Computational Methodology

Conformational analysis for the investigated BODIPY derivatives, presented in Figure 1, was performed with B3LYP-D3 and ωB97X-D functionals, together with the def2-SVP basis set, which involves the electric core potentials for heavy atoms, namely iodine, including a small core for the proper treatment of scalar relativistic effects and their recommended application for DFT approaches. The character of the stationary points was confirmed with the harmonic vibrational analysis for all the conformers. Additionally, for reference, the two BODIPY molecules unsubstituted in *meso-*position and denoted as **3** and **4** for the pristine and iodinated system, respectively, were also included into the present considerations in order to improve the method calibration and simplify the computational part relying on the conformational flexibility of the analyzed dyes. For all of the four investigated systems, the absorption spectrum in vacuum and in the selected solvents was calculated, using the vertical approach in linear response formalism. The solvent model was applied in the polarizable continuum version as implemented in Gaussian16 [47]. Due to the higher sensitivity of the excitation energy on the basis set quality, the production runs were performed with the def2-TZVP basis set [48,49,50]. Several functionals were employed for the verification of the one giving the best quality results in comparison with the experimental spectra (PBE0, CAM-B3LYP and M06-2X, according to the recommendations of Chibani, Yanai and coworkers). In the main text, M06-2X absorption wavelengths are presented, while the remaining data are given in the Supplementary Information. The strength of the chemical bonds between atoms A and B, prone to breaking, is estimated by the Wiberg bond index $I_{AB}$, defined as the sum of squares of the density matrix elements, $P_{AB}$,

(2) $$\begin{array}{c} I_{AB}=\sum_{a\in A}\sum_{b\in B}P_{ab}^{2}, \end{array}$$

due to its weak basis set dependence. [51,52,53,54] Wiberg bond indexes are estimated within the PBE0/def2-TZVP approach with the NBO program (version 3) in Gaussian16.

For the detailed description of both the intramolecular and intermolecular non-covalent interactions (NCI), the NCIPlot program was applied [55,56] for the determination of weak bonding interactions within the molecule. The type of the interactions is identified by the sign of the second eigenvalue of the electron density Hessian matrix, $\lambda_{2}$. Attractive interactions correspond to the negative values of $\lambda_{2}$, while repulsive, non-bonding contacts are characterized by $\lambda_{2}>0$.

According to the experimental measurements for the BODIPY derivatives after UV irradiation, five photodegradation mechanisms are proposed on the basis of the earlier literature reports. The computational chemistry tools are applied in order to verify the possible decomposition ways by comparison of the experimental shape of the spectra before and after irradiation with the computational absorption spectra for the hypothetical photodegradation products.

## 3. Results

### 3.1. Geometrical Aspects of BOPIDY Structure

**Figure 2:**
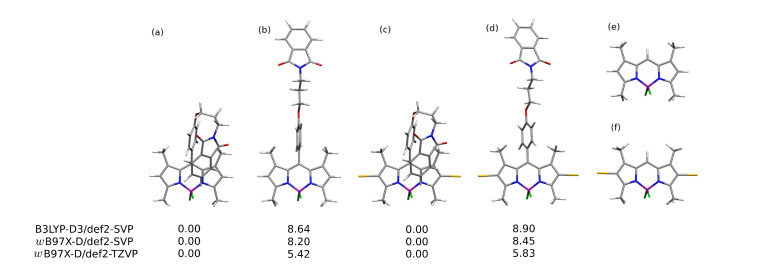
Selected BODIPY conformations analyzed in the present study: (**a**) lowest energy bent conformation of **1**, (**b**) representative unfolded conformation of **1**, (**c**) lowest energy conformation of **2**, (**d**) representative unfolded conformation of **2**, (**e**) **3** and (**f**) **4**. For **1** and **2** conformations, the relative energy in vacuum is given in kcal/mol.

**Figure 3:**
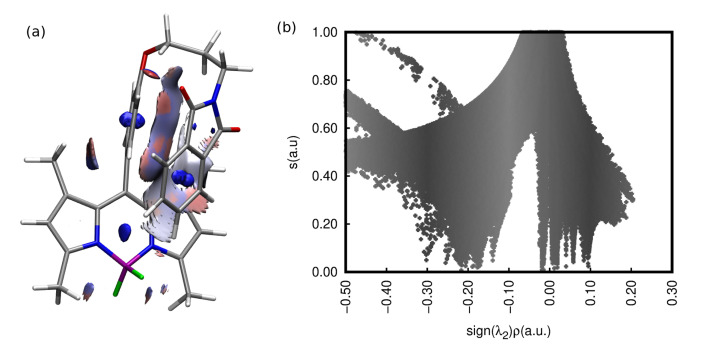
NCI analysis for the intramolecular interactions in the folded lowest-energy conformation of **1** (**a**) reduced density gradient isosurface, (**b**) reduced density gradient versus sign $(\lambda_{2})\rho$ plot.

Among the stable conformers of **1** and **2**, presented in Figure 2 and in Supplementary Information, the two general tendencies can be observed: first, the long N-phtalimide group can fold, forming the stable folded (bent) conformation, taking a maximal advantage from the mutual π−π intramolecular interaction of the long substituent with the BODIPY core. This effect is pronounced in both systems, **1** and **2**, due to the evident stacking interaction between the BODIPY core and the phthalimide substituent, independently, on the iodine presence. The visualization of these effects is presented in Figure 3 as the surfaces corresponding to the intramolecular interactions, generated in the NCIPlot program for non-covalent interactions. On the other hand, the second type of conformation, called further unfolded, expands the long substituent, forming an elongated shape that enables maximization of the exploitation of the intermolecular interactions for mutual stabilization. It should be emphasized that for the unfolded structure, the phenyl ring in the *meso-*position is aligned almost perpendicularly to the BODIPY core. This is expected to significantly affect the extent of the π-electron delocalization and, therefore, modify the photophysical characteristics of the investigated systems.

The relative ωB97X-D/def2-SVP energy of the folded structures presented in panels (a) and (c) of Figure 2 are by 8.20 and 8.45 kcal/mol more favorable than the unfolded ones (panels (b) and (d) of Figure 2), respectively for **1** and **2**. The increase in the basis set to the def2-TZVP one causes the noticeable decrease in the relative energy to 5.42 and 5.83 kcal/mol for **1** and **2**, respectively. The substitution of **1** by the iodine in the 2 and 6 positions, leading to the **2** structure, does not significantly modify the relative energy between the most stable bent and elongated (unfolded) form. These energies were compared to the phthalimide-BODIPY core interaction energy, calculated for the optimized complexes in the supermolecular manner with the B3LYP-D3/def2-SVP approach (for the corresponding structures, see Supporting Information). For the unsubstituted core **3** (compare Figure 2, panel (e)) this interaction amounts to −13.22 kcal/mol, while iodination does not change the stabilization significantly (intermolecular interaction energy of **4** equal to −13.27 kcal/mol; see Figure 2, panel (f)). Therefore, one can assume that the main contribution to the stabilization of the folded structure arises from the dispersion interaction between the aromatic parts in the molecule. The noncovalent interactions analysis in **1** confirms the importance of the intramolecular stabilizing Van der Waals forces (see Figure 3, low-density low-gradient spikes close to zero in panel (b)). A comparison of the intermolecular interactions in the bent and unfolded dyes dimers was performed in our previous work [57]. These data unequivocally show that the elongated, unfolded molecules can benefit stronger from the good matching of the aromatic parts of the molecules in dimers for effective dispersion attraction. The ωB97X-D/def2-SVP supermolecular interaction energy in the **1** unfolded dimer was estimated as almost 6 kcal/mol stronger than that for the bent dimer. A similar effect is observed for **2**; however, here, the energy difference between the unfolded and bent dimer decreases slightly to about 4 kcal/mol. Thus, it is clear that the conformation of the investigated species affects their possible aggregates to a high extent.

For the full picture, all of the localized stable conformations of **1** and **2** are presented additionally in the Supporting Information.

### 3.2. Absorption Spectroscopy

**Figure 4:**
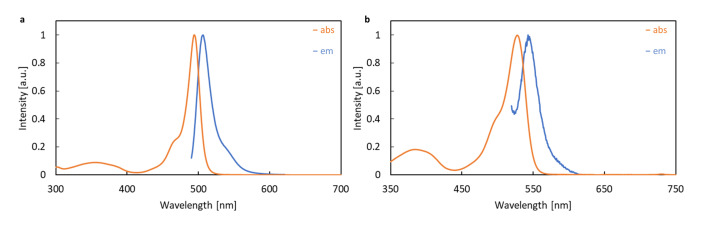
Absorption and emission spectra of (**a**) **1** ($\lambda_{ex}=480\;\mathrm{nm}$) and (**b**) **2** ($\lambda_{ex}=530\;\mathrm{nm}$) in acetonitrile.

The electronic absorption spectra of BODIPY derivatives **1** and **2** were recorded in several organic solvents, including n-hexane, toluene, ethyl acetate (EtOAc), tetrahydrofuran (THF), dichloromethane (DCM), methanol (MeOH), acetonitrile (MeCN) and dimethyl sulfoxide (DMSO) in order to confirm the stability of the investigated systems in various environments. Due to the similar shape of the spectrum in all the solvents, only the spectra recorded in acetonitrile are shown in Figure 4 and the others are given in the Supporting Information. Additionally, the corresponding wavelengths are presented in Table 1, together with their theoretical estimations.

**Figure 5:**
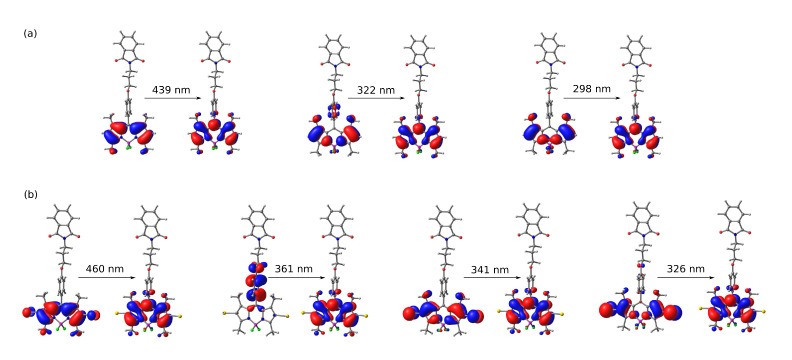
Frontier molecular orbitals of (**a**) **1** and (**b**) **2** in acetonitrile involved in most intensive transitions (M06-2X/def2-TZVP/PCM); unfolded conformations presented for clarity.

Two absorption bands were found for each compound. The sharp, intensive bands located in the range 470–520 nm and the much weaker, broad and flat bands in the range of 320–400 nm were observed for **1**. The spectrum of the iodinated derivative **2** exhibited an intensive band in the region of about 500–550 nm and a weak, flat band between 350 and 430 nm. The long-wavelength bands, around 450–550 nm, can be attributed to the $\pi ⟶\pi^{*}$ transition, while the short-wavelength flat bands at about 300–400 nm contain the contribution from the *n* orbitals of oxygen atom in the ether moiety. The molecular orbitals involved in the above transitions (depicted in Figure 5 for M06-2X/def2-TZVP/PCM approach) reveal also the mild charge transfer character for the 387 nm transition in **1** (computational estimate 322 nm) and stronger for the 413 nm transition in **2** (computational estimate 361 nm), arising from the shift of the electron density from the phenyl ring to the BODIPY core upon excitation. Moreover, one should notice the mild contribution of the iodine atoms to these singlet-singlet transitions in **2**, which is of importance in light of the photodegradation mechanisms discussed in the further part of this manuscript.

The insertion of the halogen atom at 2 and 6 positions in the core introduces the gentle absorption spectrum modification. The long-wavelength, sharp band of the absorption spectrum of **1** exhibits a maximum between 496 and 502 nm, depending on the solvent used. On the other hand, a broader, bathochromically shifted band with the maximum wavelength in the range of 528–536 nm is observed for **2**. The red-shift of the absorption band of **2** in comparison to **1** of about 30 nm can be explained by the heavy-atom effect of iodine substituted to the BODIPY core. The solvatochromic effect of the investigated dyes is tiny in size and the largest difference between the maximum of absorption in toluene and acetonitrile does not exceed 7 and 6 nm for **1** and **2**, respectively. This small bathochromic shift between the maximum absorption in different solvents is also in line with the general behavior of BODIPYs [58,59,60]. However, no correlation was found between the solvent dielectric constant ε and the wavelength at maximum absorption, $\lambda_{max}^{abs}$ and toluene, being the only solvent with the phenyl ring among the studied ones, behaves particularly, which can be noticed in Table 1. As was suggested by Piskorz et al. [22], the modification of the UV–Vis spectra of **1** and **2**, recorded in different solvents, results mainly from solvation phenomena.

The theoretical TD-DFT calculations for BODIPY dyes are known to suffer from the deficiencies in the description of the multiconfigurational systems and provide systematically underestimated absorption wavelengths [19,35,36,38,40,61]. Nevertheless, the general qualitative agreement is established between the vertical absorption estimated within the M06-2X/def2-TZVP(PCM) approach and the experimental measurements (compare Table 1): the poor dependence of the absorption bands on the solvent dielectric constant is observed in calculations, as well (compare the Supporting Information). One can also notice that the absorption spectrum of the folded (low-energy) conformer for both **1** and **2** remains red-shifted by about 6 to 7 nm with respect to the unfolded, elongated conformer and the selected conformation affects neither the qualitative discussion of the obtained data nor the comparison between the theory and experiment. The frontier orbitals involved in the most intensive transitions are presented in Figure 5. Calculations also confirm the particular behavior of a dye in toluene solution (the longest $\lambda_{theor}^{abs}$), confirming that toluene, as the only solvent with the aromatic ring included in the present study, exhibits the pronounced role of the dispersion interactions with the solute molecules.

**Figure 6:**
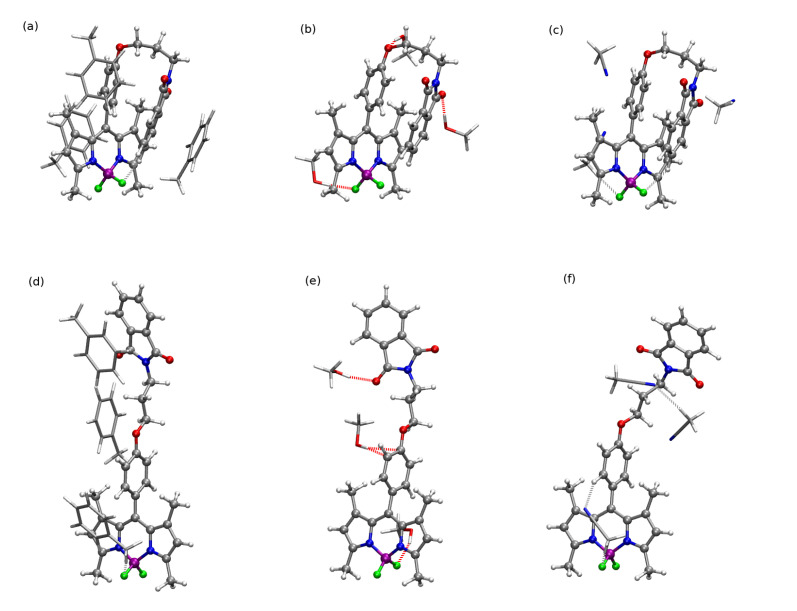
Representative optimized geometries (B3LYP-D3/def2-SVP) of the complexes of **1** with three solvent molecules for bent conformation: (**a**) toluene, (**b**) methanol, (**c**) acetonitrile. Unfolded conformation: (**d**) toluene, (**e**) methanol, (**f**) acetonitrile (dye in the CPK representation and solvent molecules as licorice; hydrogen bonds explicitely shown for 3.5 Å distance and the angle of 30 degrees.

In order to shed more light on the nature of the solute–solvent interaction, particularly in the case of the toluene solutions, theoretical calculations for the BODIPY, explicitly solvated with three solvent molecules, were performed for toluene as well as for methanol and acetonitrile within the B3LYP-D3/def2-SVP approach. The optimized complexes of **1** with three molecules of toluene, methanol and acetonitrile are presented in Figure 6. The interaction of the single toluene molecule with BODIPY is of the order of the intramolecular dispersion stabilization (−9.08 kcal/mol for **1** and −12.39 to −9.50 kcal/mol for **2**, depending on the arrangement of the aromatic rings). Aromatic rings of toluene eagerly stack either with the BODIPY core or with the phthalimide substituent. The phenyl ring in the *meso-*position of the BODIPY molecule is, on the other hand, protected by the methyl groups in 1 and 7 positions; due to sterical reasons, it does not create an effective stacked complex. On the other hand, the smaller size of the acetonitrile and methanol molecules allow to penetrate easily, even in more crowded areas, and align parallel to the phenyl ring. However, the hydroxyl group of the alcohol prefers to form strong hydrogen bonds to the ether oxygen rather than to encounter dispersion or C-H...π interactions to the phenyl ring. The interaction energy of the methanol molecules with **1** is of the order of 6–9 kcal/mol, depending on their position, and substitution with the iodine atom does not influence these interactions significantly.

### 3.3. Emission Spectra

Fluorescence emission spectra for **1** and **2** were recorded in the same series of solvents as the absorption spectra and are presented in Figure 4 for the acetonitrile solution. Table 1 summarizes the maximum emission wavelengths, Stokes shifts Δλ, and quantum yields of fluorescence ($\Phi_{F}$). **1** exhibits the intensive emission in all of the solvents. The emission maximum wavelength for **1** in DMSO is equal to 510.5 nm, Stokes shifts amounts to 0.051 eV, and the high values of $\Phi_{F}$ are observed, reaching the value of 0.43. In the non-polar solvents, namely *n*-hexane and toluene, the emission quantum yield is much lower and equal to 0.19 and 0.22, respectively. On the other hand, the iodinated BODIPY derivative **2** exhibits weak emission properties with a maximum wavelength of about 543 nm in all solvents. The quantum yield of fluorescence is very low and smaller than 0.02 in all the solvents. The substantial decrease in $\Phi_{F}$ values observed for **2** with respect to the original system **1** is in agreement with previous reports and can be ascribed to the heavy atom effect, which provides an effective intersystem crossing process from the singlet excited state to the triplet state ($S_{1}\to T_{1}$), and simultaneously decreases the fluorescence from the first excited singlet state ($S_{0}\to S_{1}$ transition), due to the diminishing of the population of this state. Thus, also, the reported solvent effects on the fluorescence quantum yield cannot be perceived as any quantitative information.

### 3.4. Photodegradation

**Figure 7:**
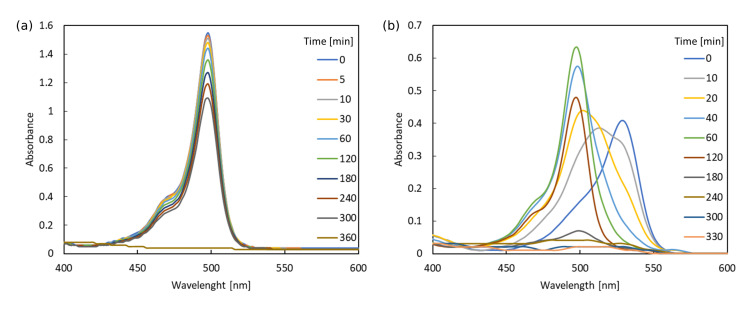
Modification of the absorption spectrum upon irradiation for **1** (**a**) and **2** (**b**) in methanol.

The photodegradation study was performed in two steps: the conventional spectroscopic analysis of the absorption spectrum modification upon sample irradiation was followed by the detailed chromatographic investigation. The spectra registered upon irradiation of both samples in methanol as the photochemically inert solvent are presented in Figure 7. The spectroscopic studies showed that the photodegradation process for compounds **1** and **2** proceeds in significantly different ways, both in the shape of the absorption spectra, timescale and kinetics. However, the common feature for both cases is that the long exposition times lead to the diminishing of the intensive absorption bands ascribed to the $\pi ⟶\pi^{*}$ transitions in the range of about 496 for **1** and 529 nm for **2** as well as the less intensive $n⟶\pi^{*}$ bands in the 300–400 nm range. The modification of the absorption spectrum upon irradiation occurring slowly enough in methanol in order to notice all the subsequent changes in time allow to observe first the relatively fast decrease in the intensity of the long wavelength signal at 529 nm and simultaneously, the gradual uplift of the shorter wavelength band at 500 nm. The analogous changes in acetonitrile are too fast to notice both signals modification and rather a cumulative picture consisting of the band shift only is observed. Therefore, in the present discussion, only the photodegradation in methanol is presented.

After the long irradiation, only the small intensity strongly hypsochromically shifted signals remain in both spectra, which indicate the photodegradation of the original compounds. Nevertheless, this simple approach to photodegradation investigation does not allow to precisely ascribe the obtained products and determine the detailed mechanism of the occurring changes. Therefore, on the next step, the photodegradation process was investigated, using HPLC chromatography with a DAD detector. This allows for the separation of the individual photodegradation products after successive exposure times and simultaneous registration of the absorption spectra of photoproducts in the UV–Vis range. Such an approach provides the information of the number of the observed photoproducts, their concentration during the irradiation process and their absorption spectrum, which enables to ascribe their structure and thus establish the mechanism of the process.

**Figure 8:**
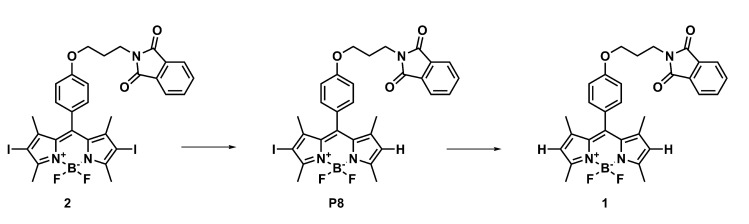
Photodegradation mechanism I: the iodine detachment from **2**.

**Figure 9:**
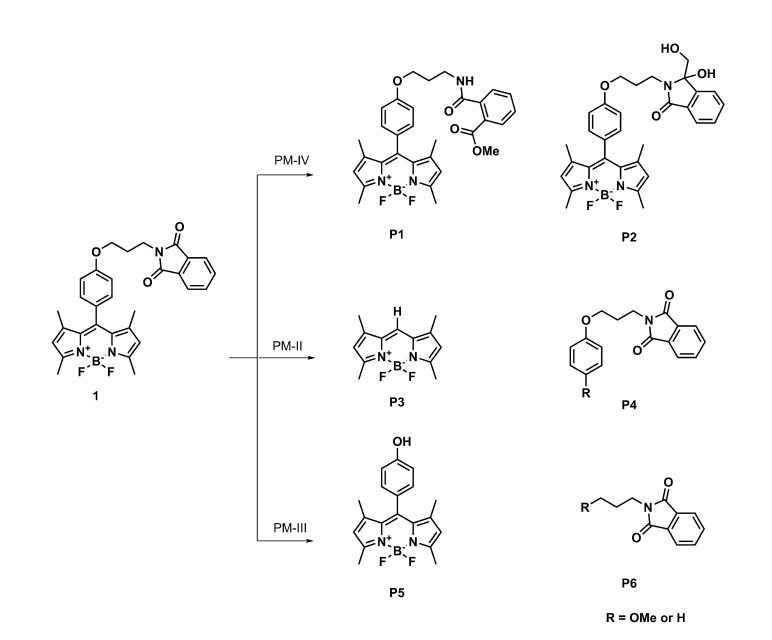
Pathways of photochemical changes leading to photoproducts with an absorption maximum of 500 nm and their fragments (photodegradation mechanisms II–IV).

**Figure 10:**
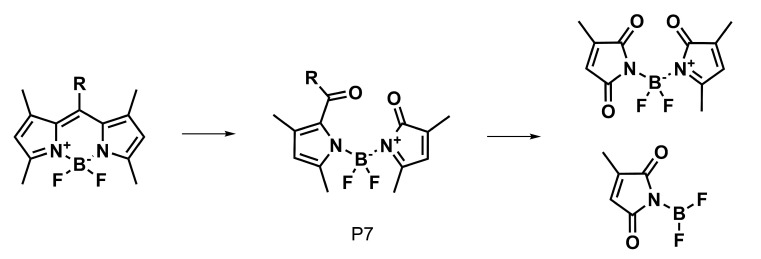
Photooxidation of BODIPY according to photodegradation mechanism V.

The strength of the bonds in the BODIPY molecule and the commonly encountered photodegradation mechanisms in similar systems [30] indicate that several possible ways of photodegradation should be taken into account in the present study. Five possible mechanisms are considered here: (1) the photodetachment of iodine atoms leading from **2** to **1** photodegradation products (this way is denoted further as the photodegradation mechanism I, PM-I, Figure 8), (2) the photodetachment of the *meso-*substituent by the BODIPY core-phenyl ring C-C bond breaking [30] (photodegradation mechanism II, PM-II, Figure 9), (3) C-O bond photolysis in the *meso-*substituent (photodegradation mechanism III, PM-III, Figure 9), (4) the phthalimide moiety destruction (photodegradation mechanism IV, PM-IV, Figure 9), and finally (5) the photooxidation of the BODIPY core as suggested by Mula et al. [30] (photodegradation mechanism V, PM-V, Figure 10). The observed photoproducts are further on denoted as PX, where X is the subsequent number roughly in agreement with the order of their appearance in Figure 8, Figure 9 and Figure 10.

**Figure 11:**
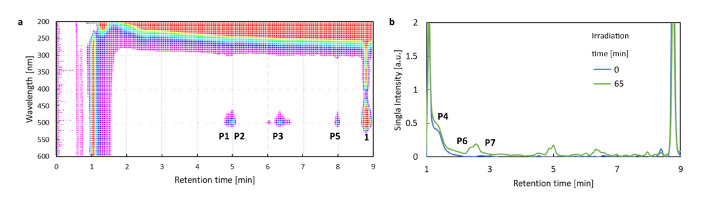
Chromatograms indicating the multiple photodegradation products appearing upon irradiation in methanol: (**a**) 2D chromatogram (absorption wavelength vs. retention time) for 1 after irradiation time 80 min (**b**) chromatogram for compound **1** after irradiation time 65 min in comparison with chromatogram for mixture before irradiation (the signal was monitored at wavelength 254 nm).

For pristine system **1**, the retention time was equal to 8.77 min (Figure 11, panel (a)). After 5 min of irradiation, a decrease in the concentration of **1** was observed. After 20 min of irradiation, the appearance of five new peaks for the photodegradation products of **1**, denoted as P1, P2, P3, P4, was observed. The products P1, P2, P3 exhibit the absorption maximum at 500 nm, which indicates that the BODIPY core remains intact upon the photochemical changes. The P4 photoproduct, on the other hand, absorbs in the UV region and can, thus, be the fragment of **1**. After the irradiation time t = 35 min, the chromatogram shows new peaks originating from the product P5 absorbing in the UV region. Starting from this moment, another colorless compound P7 appears, which may be a product of the disintegration of the BODIPY ring.

**Figure 12:**
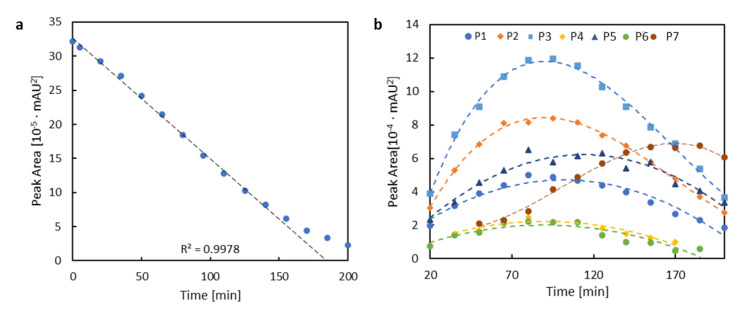
(**a**) Changes in concentration of **1** together with the straight line fit for the introductory stage (estimated rate constant k = 10^−11^ mol/(dm^3^·s)), (**b**) modification of concentration of the photodegradation products of **1** in methanol expressed as the change of the area of the chromatographic peak.

During the irradiation time from t = 0 min to t = 150 min, the concentration of **1** decreases linearly, which can be seen in Figure 12a). This indicates that the photodegradation process for **1** in dilute solution follows the zeroth-order kinetics. The analysis of the composition of the mixture after irradiation shows that P1–P7 products reach their highest concentration after the irradiation time of 80 min (Figure 12b). After this time, the concentration of these substances—estimated as the peak area in the chromatogram—decreases upon irradiation. Only the P7 product increases its concentration almost throughout the entire process, which suggests that as a result of the photochemical changes, the chemical bonds may break with the dissociation of a part of the molecule that absorbs only UV radiation.

**Figure 13:**
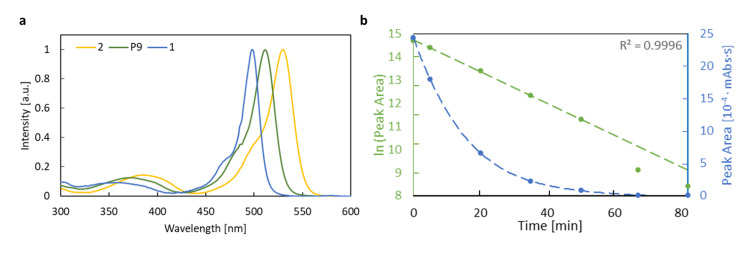
Absorption spectrum for the products of photodegradation in methanol after exposure time t = 20 min (**a**), the correlation between the area of the chromatographic peak (blue) and the relationship between the natural logarithm of the area of the chromatographic peak A (green) and the time (**b**) (estimated rate constant k = 1.2·10^−4^ $s^{-1}$).

A different course of the photodegradation process was observed for **2** (chromatograms not shown). Already after 5 min of irradiation, the peak of the substance P9 occurs in the chromatogram with the maximum absorption at 512 nm. After the irradiation time t = 20 min, another peak appears in the chromatogram, with $\lambda_{max}$ = 500 nm. The retention time for this substance is $t_{R}$ = 8.03 min. Comparing the retention times for the product appearing in Figure 11 and the experimental results in the spectroscopic part of the study (Figure 7), it can be deduced that it is the deiodinated system **1**. Thus, **1** is one of the products of photodegradation that occur under the influence of the irradiation of **2** during fragmentation, according to the photodegradation mechanism I (PM-I), presented in Figure 8. The corresponding absorption spectra of the substances formed as a result of the photochemical changes during irradiation t = 20 min are presented in panel (a) of Figure 13. After the irradiation time t = 35 min, the signals from the subsequent photoproducts appear. One of them exhibiting the maximum absorption at 500 nm can be identified as P7, occurring also in the photodegradation pathway for **1**. Additionally, it is important to note that the P7 (also observed for pristine **1** photodegradation) and P9 products absorb only in the UV range.

For **2**, the kinetics of the photochemical reaction in the first 50 min of irradiation is determined. Since the change in concentration of the investigated substances is directly proportional to the area of the chromatographic peak A (Figure 13), the logarithm of these quantities are also proportional to each other. The relationship between the natural logarithm of peak A area and the exposure time is linear (Figure 13). Based on this, it can be concluded that the photochemical changes for **2** follow first-order kinetics. Additionally, the decrease in the concentration for the starting compounds is much greater for **2** than for **1**, which can be explained when the photooxidation process is assumed. It is rough to explicitly prove with the selected techniques applied in the present study; however, it can justify the different degradation rates for iodinated and pristine derivatives. Namely, the iodinated **2** system is more prone to the attacks of the reactive oxygen species, generated by **2** in higher amounts than by **1**, due to the heavy atom presence and thus the stronger intersystem crossing (singlet oxygen generation quantum yield determined by Piskorz et al. [22] for **2** in methanol is equal to 0.97, while for **1** it accounts only for 0.02). Furthermore, the formation of singlet oxygen induces photo-oxidation processes that can lead to a wide variety of products that are not recorded by the selected chromatography method. Photo-oxidation may also explain the lower amount of products with an absorption maximum at 496 nm, observed for **2** than for **1**. If such products are obtained, their concentration may not be high enough for their detection.

## 4. Discussion

One can see that besides the strong confirmation of the iodine detachment in the first stage of the photodegradation (PM-I, Figure 8) by the experimental techniques, the complexity of the analyzed processes makes it not easy for undoubtful confirmation. Therefore, for the hypothetical mechanisms also, the theoretical calculations were performed in order to confirm the reason of the absorption decrease over long irradiation times. Due to the complicated pathways of the possible photofragmentation processes, the theoretical analysis was limited to the investigation of the absorption spectrum of the photodegradation products, which are expected to be observed experimentally after the irradiation [62]. This is of importance for the possible biomedical application of the analyzed BODIPY compounds, which could, in general, decompose with the creation of any products detrimental for living organisms.

The Wiberg bond indexes, which allow to estimate the weakest bonds, were calculated for **1** and **2** bent and unfolded conformations with the PBE03/def2-TZVP approach in methanol and are presented in Table 2. According to these results, one could consider reliable all of the above-mentioned photodegradation mechanisms, namely, the iodine detachment, the detachment of the fluorine atoms, the C20–O21 or O21–C22 bond breaking in the ether bridge of the molecule, the disconnection of the phthalimide group by the C24–N25 bond cleavage and the phthalimide heterocyclic ring opening by N25–C26 bond breaking. All of these bonds are characterized by the Wiberg bond indexes close to or lower than 1.0.

**Figure 14:**
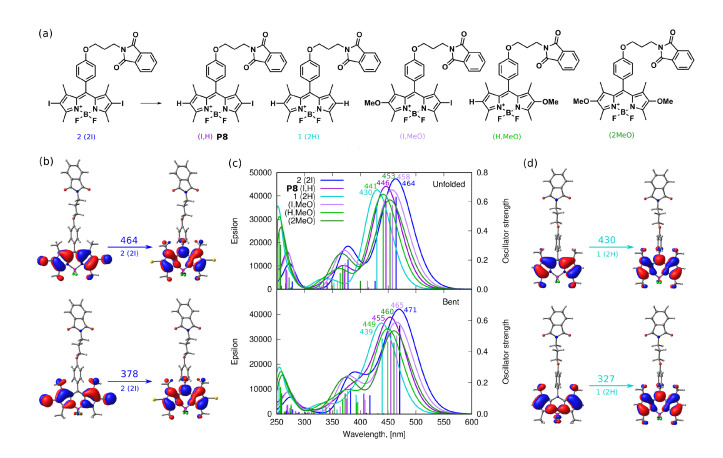
Photodegradation mechanism, I—Iodine detachment: (**a**) hypothetical photodegradation products, (**b**) molecular orbitals involved in the most intensive transitions for the initial iodinated system **2**, (**c**) the PBE0/def2-TZVP(methanol) theoretical absorption spectra for unfolded and bent conformation, (**d**) molecular orbitals involved for the most intensive transitions of **1** deiodinated system (in the key, in parenthesis, the substituents in 2 and 6 positions are explicitly given).

First of all, the iodinated derivative **2** was assumed to lose the iodine atoms either in a concerted or rather stepwise manner, as presented in Figure 8 (PM-I). The dissociation of the iodine atoms for the processes occurring in methanol can also coexist with the methoxy radical formation. In such a case, the BODIPY core, willing to react with anything in close proximity, can possibly catch and bind the methoxy moiety in place of the iodine substituent. The absorption spectrum for these considered photodegradation products of PM-I were roughly estimated with the computational tools for the two conformers (bent and unfolded) and are presented in Figure 14 together with the corresponding molecular orbitals for **1** and **2**. One can clearly see that the computational results indicate the hypsochromic shift of the maximum absorption wavelength by about 34 nm when going from the iodinated **2** to unsubstituted **1** molecule for the elongated (unfolded) conformers. The corresponding shift for the lowest energy bent conformations amounts to 32 nm in methanol. Additionally, the second band calculated for **1** at 378 nm significantly decreases for the unsubstituted unfolded molecule **2**. The dominating orbitals contributing to these excitations are localized at the BODIPY core with only minor influence arising from iodine atoms. The presence of the metoxy group arising from the probable radical reactions involving the solvent molecules in the BODIPY core only moderately affects the calculated spectrum; therefore, one can expect that this is not the radical solute–solvent interaction crucial for the observed absorption changes upon irradiation. Taking into account the agreement of the initially recorded modification of the experimental spectrum, namely, the signal shift after the irradiation times up to 60 min of the order of 30 nm (compare Figure 7), one can assume that the detachment of the iodine atoms from **1** is indeed the first step of the observed photodegradation. For this reason, in the following part of the discussion of photodegradation mechanisms, only the unsubstituted systems (with no iodine) are included in the considerations.

**Figure 15:**
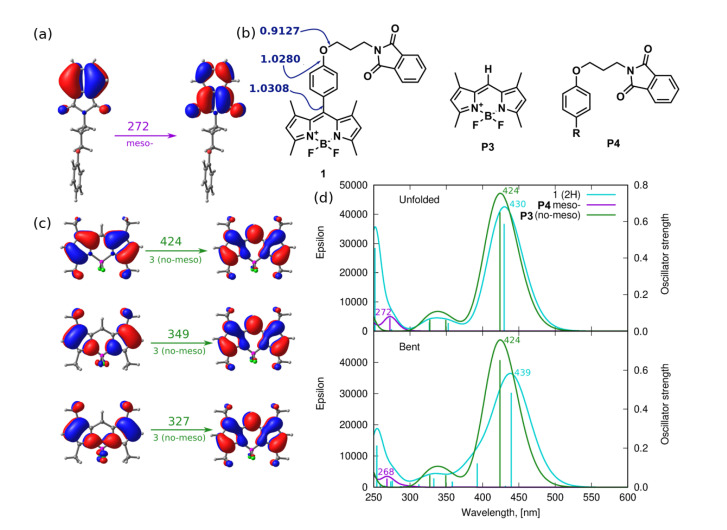
Photodegradation mechanism II—*meso-*substituent detachment: (**a**) molecular orbitals involved in the most intensive transitions for the detached *meso-* substituent, (**b**) hypothetical degradation products with the corresponding Wiberg bond indexes in the C–O–C fragment of unfolded **1** (in navy blue), (**c**) molecular orbitals involved in the most intensive transition of the photodegradation product **3**, (**d**) the theoretical absorption spectra for the hypothetical PM-II products (where photodegradation product P3 is identical with **3** unsubstituted non-iodinated BODIPY core).

The calculated Wiberg bond indexes for the C8–C17 bond linking the BODIPY core with the *meso*-substituent reveal relatively weak bonding of this fragment (1.0308 for unfolded **1** structure); therefore, one of the considered photodegradation mechanisms, depicted in Figure 9, involves this particular decomposition pathway (PM-II). The calculated absorption spectra for the *meso*-substituent and its BODIPY core counterpart (denoted here as **3**) is presented in panel (b) of Figure 15. One is aware that the structure of the investigated *meso*-substituent bearing the phenyl ring and the phthalimide moiety bound by the aliphatic chain and ether oxygen does not exhibit absorption in the visible region and only weakly depends on the conformation of the chain. Therefore, its presumptive presence in the photodegradation products mixture is of no importance for the recorded absorption spectra after irradiation (see Figure 7). The detachment of the *meso*-substituent causes the hypsochromic shift of the most intensive absorption band by about 6 and 15 nm for the unfolded and bent conformers, respectively.

**Figure 16:**
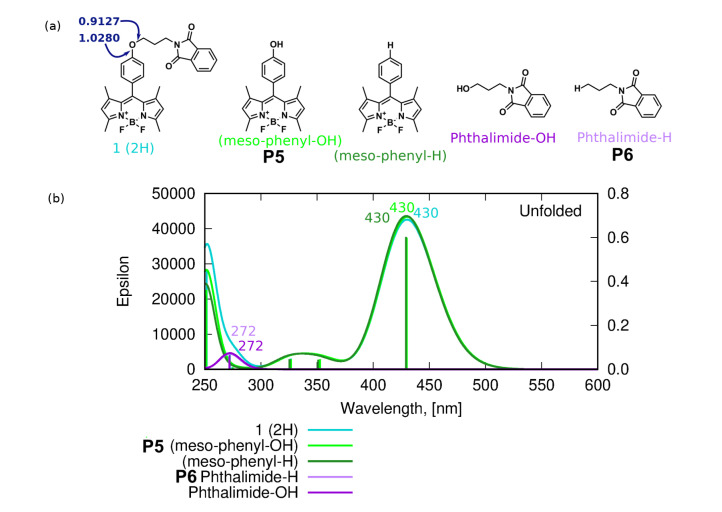
Photodegradation mechanism III–C–O bond photolysis in the *meso-*substituent: (**a**) hypothetical degradation products with the corresponding Wiberg bond indexes in the C–O–C fragment in unfolded **1** (in navy blue), (**b**) the theoretical absorption spectra for the hypothetical PM-III products.

The photodegradation mechanism III, investigated in the present study (Figure 9), includes the disconnection of the long chain substituent, due to the relatively weak C–O bond presence that is often prone to photolysis upon UV–Vis irradiation. The possible reaction and the theoretical absorption spectrum of the assumed products are presented in Figure 16. The considered C–O bond breaking either between the phenyl ring and ether oxygen (C20–O21 Wiberg bond index 1.0280 for unfolded **1**) or between the ether oxygen and phthalimide group (O21–C22 Wiberg bond index 0.9127) leads to the degradation products with the absorption spectra shifted negligibly with respect to the original **1** molecule, with no observable intensity modification. Therefore, one can assume again that it is not this photodegradation path that is responsible for the experimentally recorded changes of the absorption spectra upon the long irradiation presented in Figure 7, namely, the significant decrease of the maximum absorption wavelength together with the spectrum hypsochromic shift.

**Figure 17:**
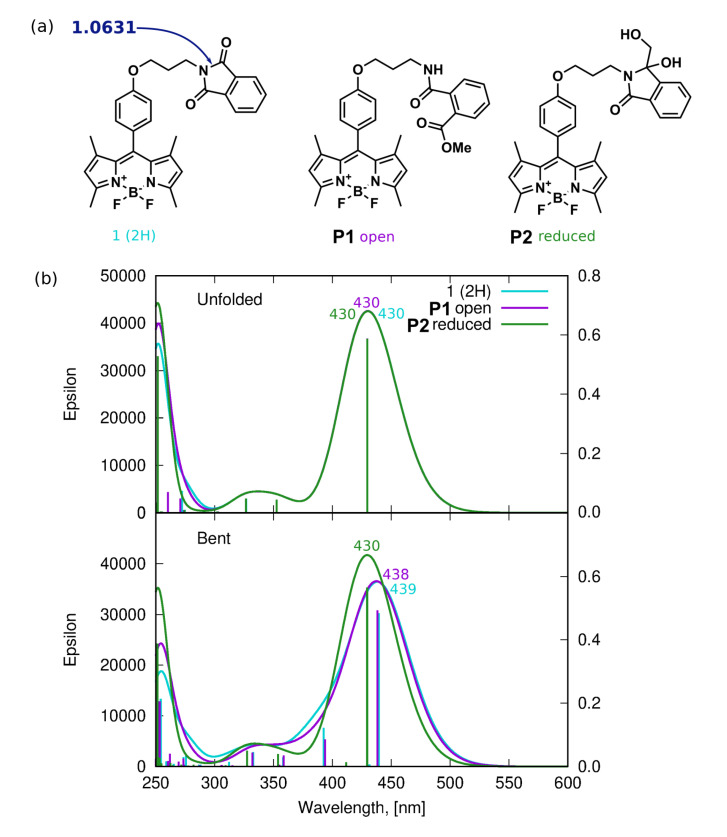
Photodegradation mechanism IV—phthalimide ring opening: (**a**) hypothetical products with Wiberg bond order for breaking the bond in the phthalimide moiety given in navy blue, (**b**) the theoretical absorption spectra for the hypothetical PM-IV products.

Another possibility of the influence of UV irradiation is the destruction of the phthalimide moiety itself, presented in Figure 9 (photodegradation mechanism IV, PM-IV). The Wiberg bond indexes for the N25–C26 bonds likely to break in the phthalimide moiety are, indeed, close to 1.0 (see Table 2), legitimizing this mechanism. The corresponding spectra of hypothetical products of this process presented in panel (b) of Figure 17 indicate that the only modification in the spectrum is the small blue-shift for the bent conformer (439 vs. 430 nm). Therefore, also this pathway of photodegradation of the investigated BODIPY dyes seems to not introduce the changes observed experimentally for the mixture irradiated for a long time (Figure 7).

**Figure 18:**
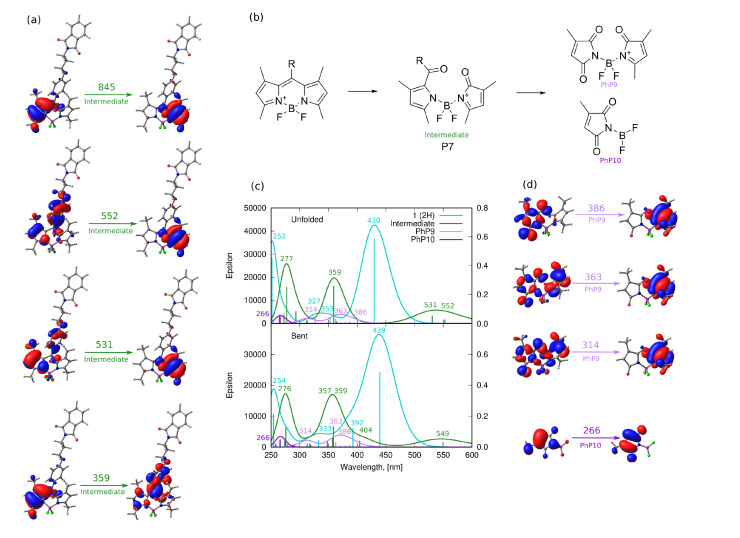
Photodegradation mechanism V–BODIPY core photooxidation ring opening: (**a**) molecular orbitals involved in the most intensive transitions for intermediate product, (**b**) hypothetical photodegradation products, (**c**) the theoretical absorption spectra for the hypothetical PM-V products, (**d**) molecular orbitals involved in the most intensive transitions for the final products of photo-oxidation.

The remaining possible, and, at the same time, the strongest, structural modification deeply affecting the molecule character and thus the absorption of the BODIPY dyes is connected to the boron heterocyclic ring breaking (photodegradation mechanism V, PM-V). This pathway was suggested by Mula et al. [30] with the application of mass spectrometry tools. It is expected to occur via the photo-oxidation mechanism as presented in Figure 18, panel (b). One can assume that this process really can lead to the diminishing of the strong absorption bands at 450 and 370 nm for the original compounds since these transitions involve molecular orbitals localized just on the BODIPY core rings. Indeed, the theoretically estimated absorption spectra for the final photodegradation products in this mechanism exhibit weak or no absorption in the 350–450 nm range and the intensity of any signals is significantly diminished with respect to the original BODIPY dyes. The initial step of the photo-oxidation of the central BODIPY ring, leading to its opening in the *meso*-position, produces the photoproduct of the weak absorption at 425, 394 and 341 nm, while the further total devastation of the system by the B4–N11 bond breaking (Wiberg bond index 0.6871 for **1** unfolded, being actually the weakest bond in the whole molecule) to the generation of the single substituted succinimide, brings the only weak absorption band to 314 nm. All of the remaining bands in the spectrum vanish for this small system. All of these theoretical calculations together with the experimental observations of the irradiated system spectra lead to the rationalization of the initial assumption that the modification of the spectrum upon UV–Vis irradiation is a stepwise process, including the detachment of the iodine atoms in the first stage, probable *meso*-substituent detachment (hard to confirm directly with spectroscopic techniques, due to the negligible changes in the absorption spectrum) and finally, the photo-oxidation of the BODIPY core in the last stage.

## 5. Conclusions

The present study devoted to the photostability of the two BODIPY derivatives, synthesized previously for bacteria photoinactivation, combines the theoretical and chromatographic approaches. The two systems containing the phthalimide moiety in the *meso*-position differ by the absence/presence of the iodine substituents in 2 and 6 positions for **1** and **2**, respectively. The *meso*-position occupied by the aromatic system and connected with the BODIPY core by the long linker allows the adaptation of different structures, benefiting either from the intramolecular dispersion interaction for bent molecules, or from the maximized intermolecular π−π stacking for elongated ones. The iodine presence in the structure only mildly affects the geometrical properties. Among the five photodegradation mechanisms considered on the basis of the available literature reports, the absorption measurements after UV irradiation suggested that the first stage consists of iodine atoms detachment. This is confirmed by the chromatographic investigation, which further allow to determine the structure of the main photodegradation products and their concentration changes during the irradiation. Despite the strong structural similarity of both structures **1** and **2**, the photodegradation process in both systems differs to a high extent and discrepancies are observed in the shape of the spectra upon irradiation, the timescale of the fragmentation and the kinetics of the reactions. The DFT verification of the absorption spectra of the hypothetical irreversibly destructed photodegradation products seems to inevitably confirm that the total diminishing of the strong absorption bands upon the irradiation arises from the destruction of the BODIPY core by the photo-oxidation process. This observation remains in agreement with the larger singlet oxygen quantum yield in the case of **2**, which enables easier oxidation of the BODIPY core than in **1**.

## Figures and Tables

**Table 1:** Photophysical and photochemical characteristics of **1** and **2** at various solvents characterized by their dielectric constant ε: maximum absorption wavelength $\lambda_{max}^{abs}$ [nm], maximum emission wavelength $\lambda_{max}^{em}$ [nm], Stokes shift Δλ [eV], fluorescence quantum yield $\Psi_{F}$. For comparison, also the $\lambda_{theor}^{abs}$ [nm] estimated with the M06-2X/def2-TZVP vertical approach in PCM for the unfolded and bent conformations are presented.

Solvent	ε	$\lambda_{\max}^{\mathrm{abs}}$	$\lambda_{\max}^{\mathrm{em}}$	Δλ	$\Phi_{F}$	$\lambda_{\mathrm{theor}}^{\mathrm{abs}}$	$\lambda_{\mathrm{theor}}^{\mathrm{abs}}$
						Unfolded	Bent
**1**							
Hexane	1.8819	500	508.5	0.041	0.19	442	448
Toluene	2.3741	502	512.0	0.048	0.22	446	452
EtOAc	5.9867	498	506.5	0.042	0.56	441	448
THF	7.4257	500	509.0	0.044	0.39	442	449
DCM	8.93	500	509.5	0.046	0.40	442	449
MeOH	32.613	496	506.5	0.052	0.35	438	446
MeCN	35.688	496	505.5	0.047	0.36	439	446
DMSO	46.826	500	510.5	0.051	0.43	442	449
**2**							
Hexane	1.8819	532	543.0	0.047	0.02	454	469
Toluene	2.3741	536	549.5	0.057	0.01	458	473
EtOAc	5.9867	530	543.5	0.058	0.01	452	468
THF	7.4257	532	546.5	0.062	0.01	453	468
DCM	8.93	532	545.5	0.058	0.02	453	469
MeOH	32.613	529	543.0	0.060	0.01	449	465
MeCN	35.688	528	544.5	0.071	0.01	450	
DMSO	46.826	536	546.0	0.042	0.01	452	468

**Table 2:** Wiberg bond indexes for **1** and **2** estimated with PBE0/def2-TZVP approach in methanol (atom numbering presented for the exemplary structure).

	Bond	1		2	
		Bent	Unfolded	Bent	Unfolded
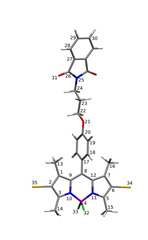	BODIPY core				
	B4–N11	0.6852	0.6871	0.6764	0.6781
	B4–F32	0.7187	0.7241	0.7255	0.7300
	C8–C9	1.3325	1.3207	1.3246	1.3156
	C8–C17	1.0091	1.0308	1.0173	1.0328
	C7–C16	1.0575	1.0565	1.0558	1.0558
	C5–C15	1.0590	1.0586	1.0585	1.0581
	C6–I/H34	0.9140	0.9129	1.0235	1.0249
	*meso*-substituent				
	C20–O21	1.0413	1.0280	1.0464	1.0307
	O21–C22	0.9183	0.9127	0.9168	0.9115
	C24–N25	0.9593	0.9509	0.9594	0.9508
	N25–C26	1.0598	1.0631	1.0597	1.0643
	C26–O31	1.7237	1.7123	1.7239	1.7115

## Data Availability

Additional data are available as the Supplementary Information.

## Supplementary Materials

The following are available online at https://www.mdpi.com/article/10.3390/ijms22136735/s1.
