# Supplementary material for: Photochemical Properties and Stability of BODIPY Dyes
Source: Int J Mol Sci. 2021 Jun 23;22(13):6735. doi: 10.3390/ijms22136735 (PMC8267640; doi:10.3390/ijms22136735)
Supplement: Supplementary file 1 [file ijms-22-06735-s001.zip › ijms-1242104-supplementary.pdf]

Supporting Information: Photochemical properties and stability of BODIPY dyes

Patryk Rybczyński,† Aleksander Smolarkiewicz-Wyczachowski,† Jarosław Piskorz,‡ Szymon Bocian,† Marta Ziegler-Borowska,† Dariusz Kędziera,† and Anna Kaczmarek-Kędziera*,†

†*Faculty of Chemistry, Nicolaus Copernicus University in Torun, Gagarina 7, 87–100 Toruń, Poland*

‡*Department of Inorganic and Analytical Chemistry, Poznan University of Medical Sciences, Grunwaldzka 6, 60-780 Poznan, Poland*

E-mail: teoadk@chem.umk.pl

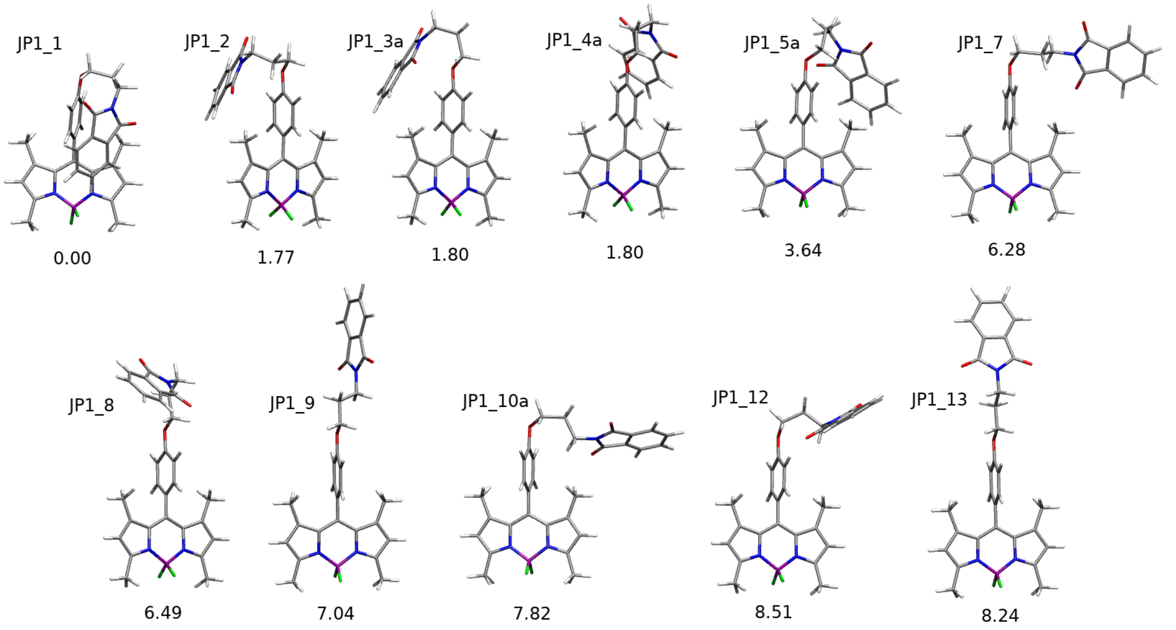

Figure S1: Optimized structures of the localized stable minimum conformations of **1** and their relative energy

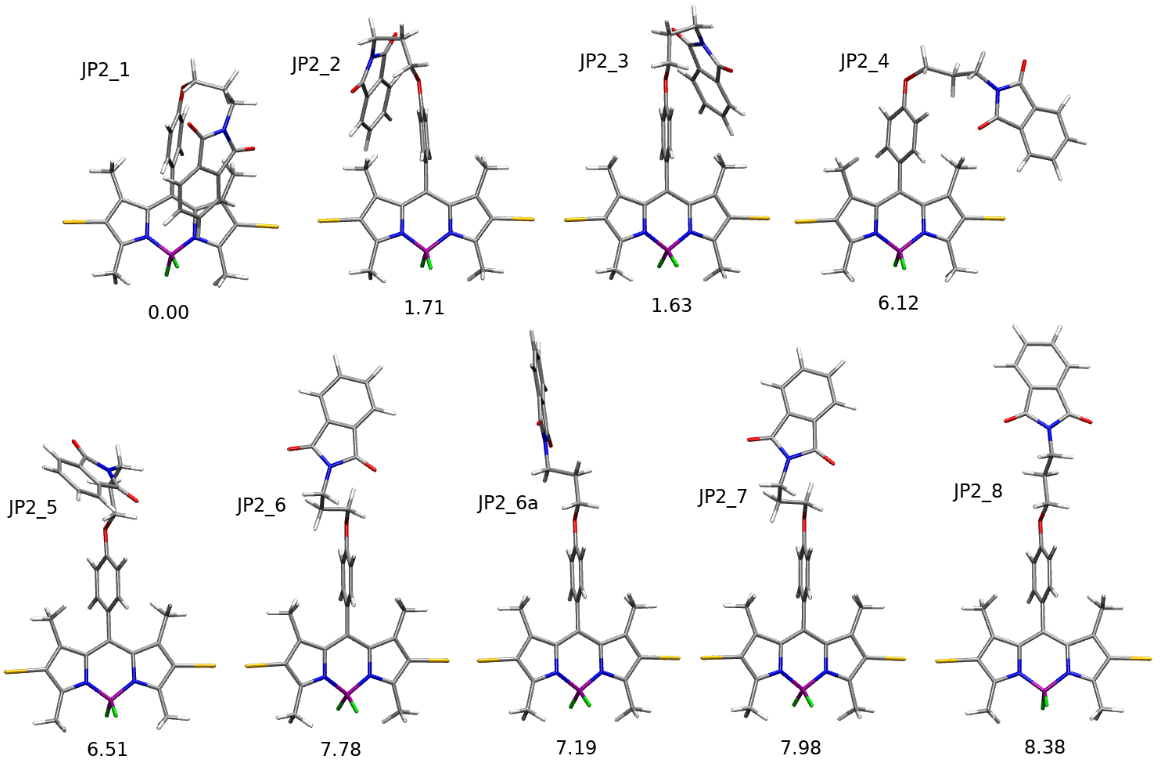

Figure S2: Optimized structures of the localized stable minimum conformations of **2** and their relative energy

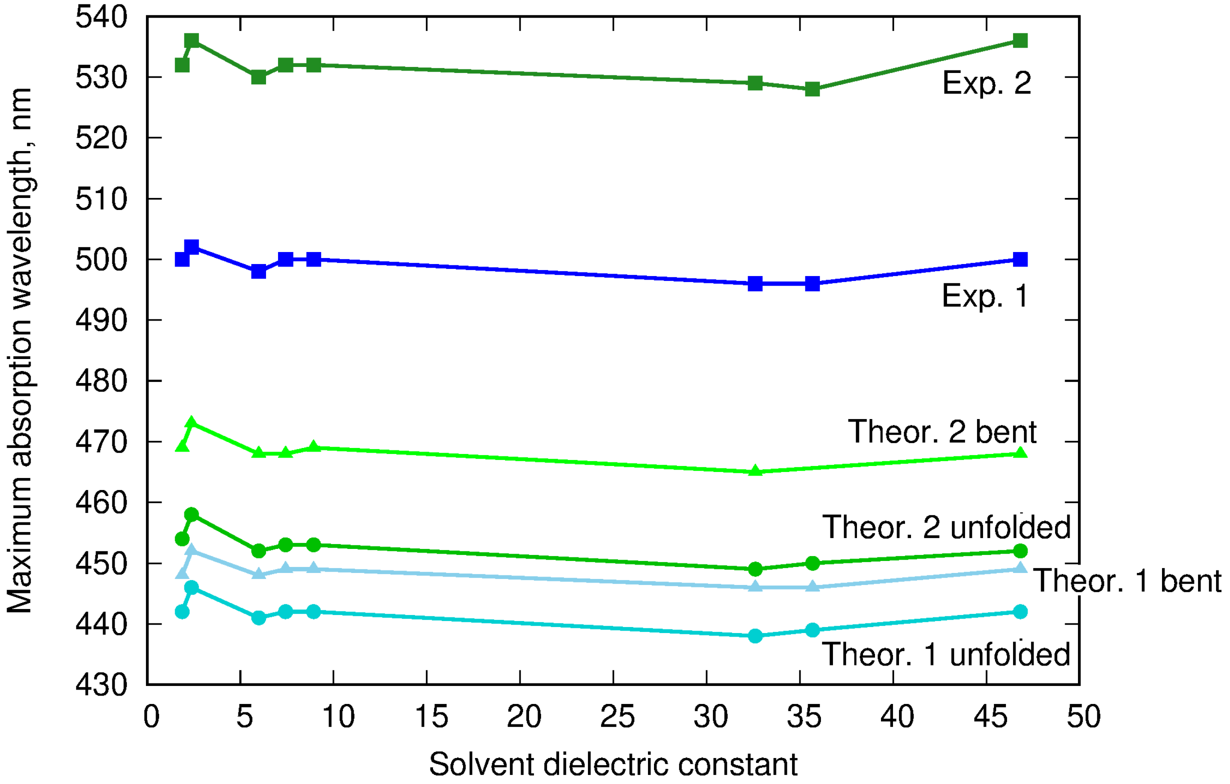

Figure S3: Experimentally measured maximum absorption wavelength [nm] versus solvent dielectric constant in comparison with the theoretical data for bent and unfolded conformers of **1** and **2** (M06-2X/def2-TZVP/PCM, vertical approach)

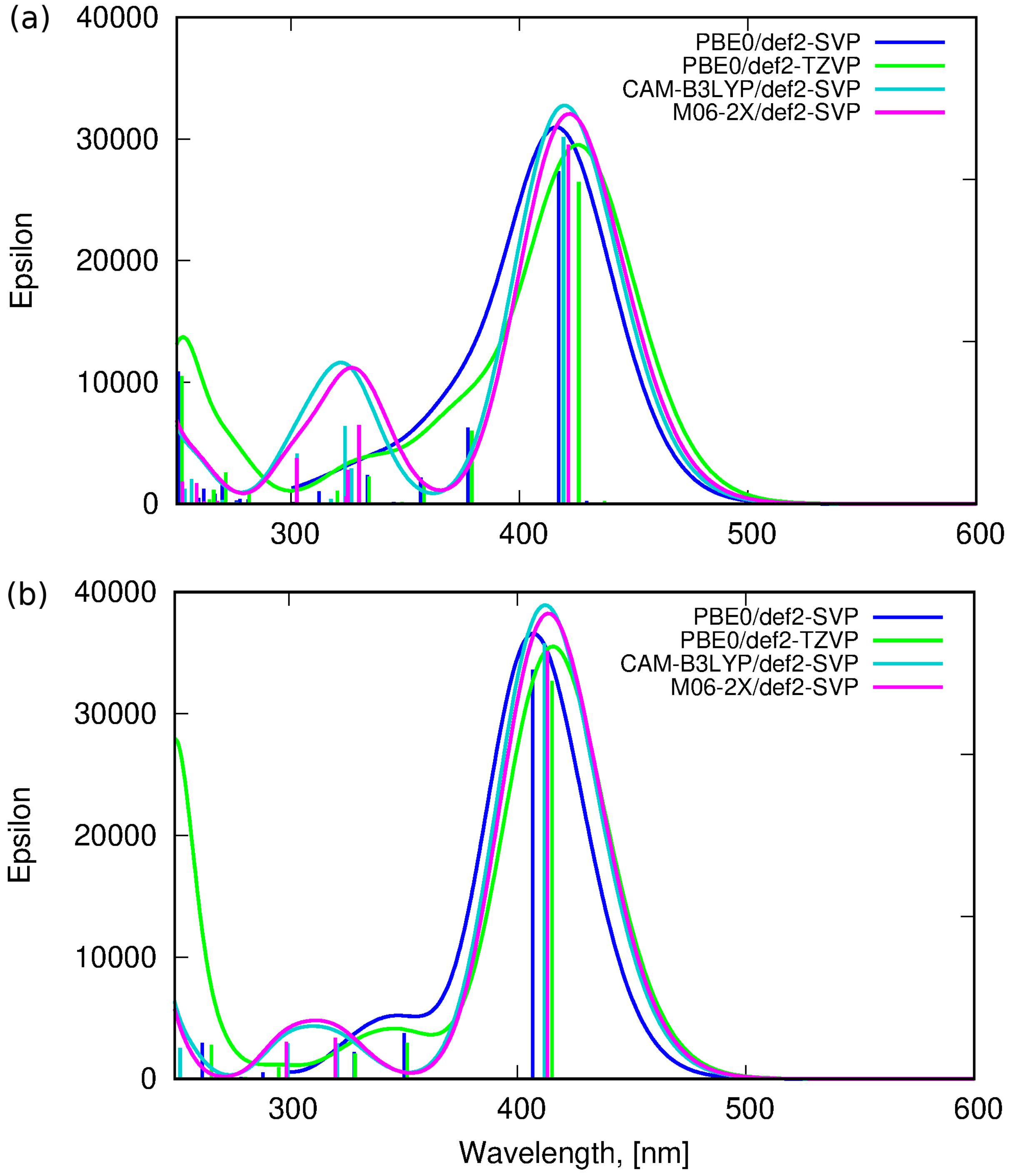

Figure S4: Estimation of the effect of functional and basis set choice for the quality of the vertical absorption spectrum in vacuum for **1** in (a) bent, (b) elongated conformation (one can notice the negligible influence of the functional and basis set for the most intensive absorption band)

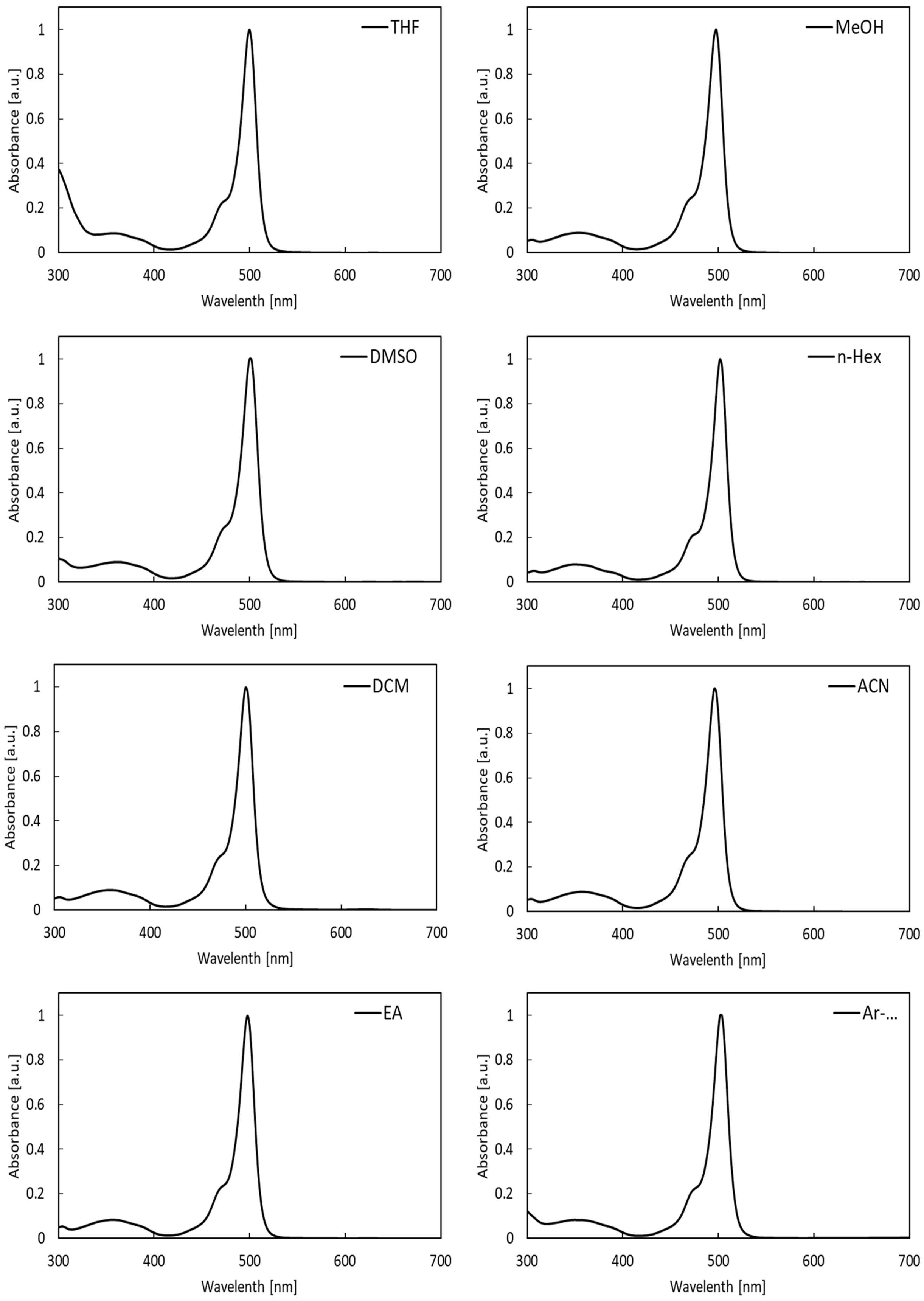

Figure S5: Absorption spectrum in different solvent for **1**

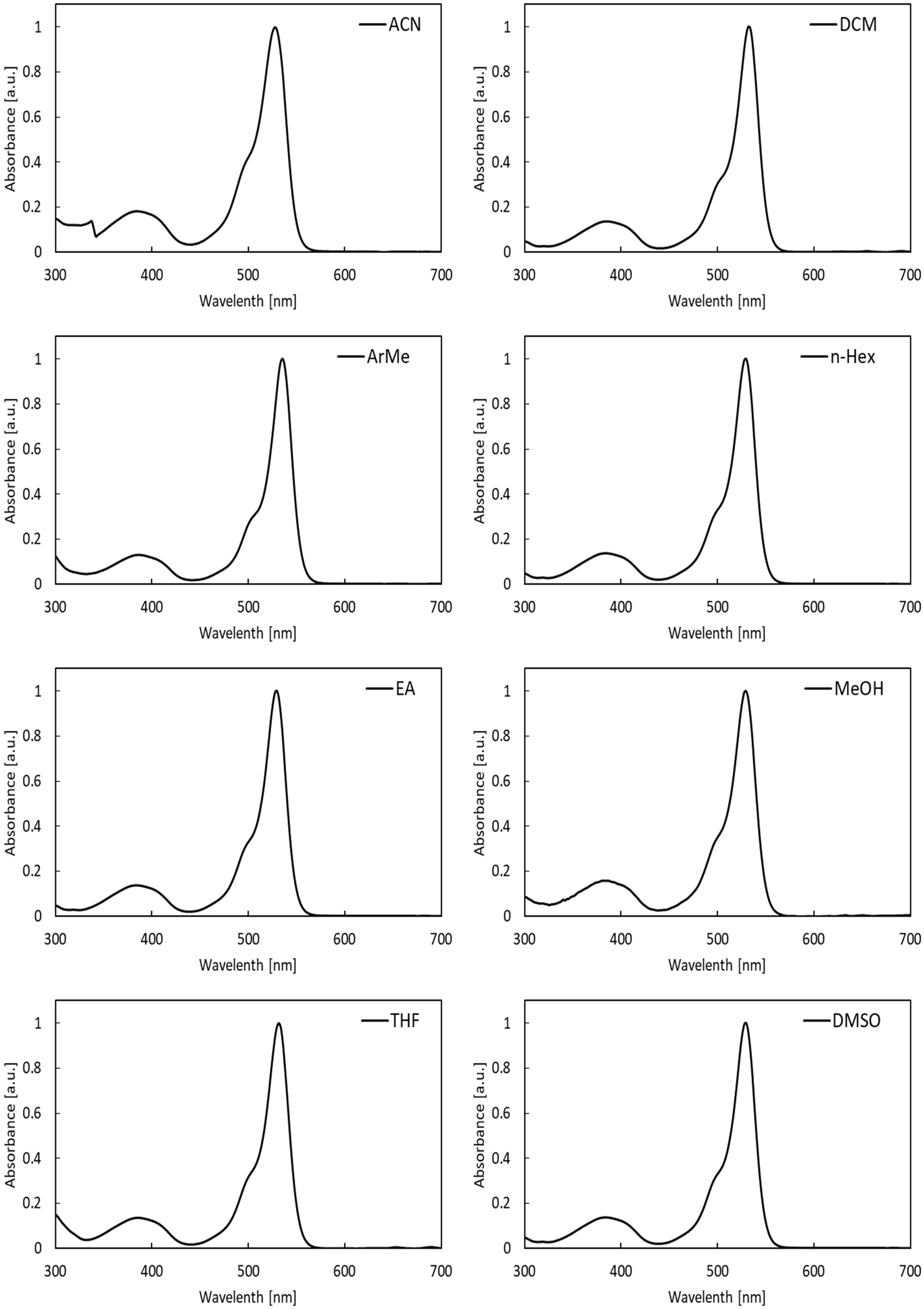

Figure S6: Absorption spectrum in different solvent for **2**

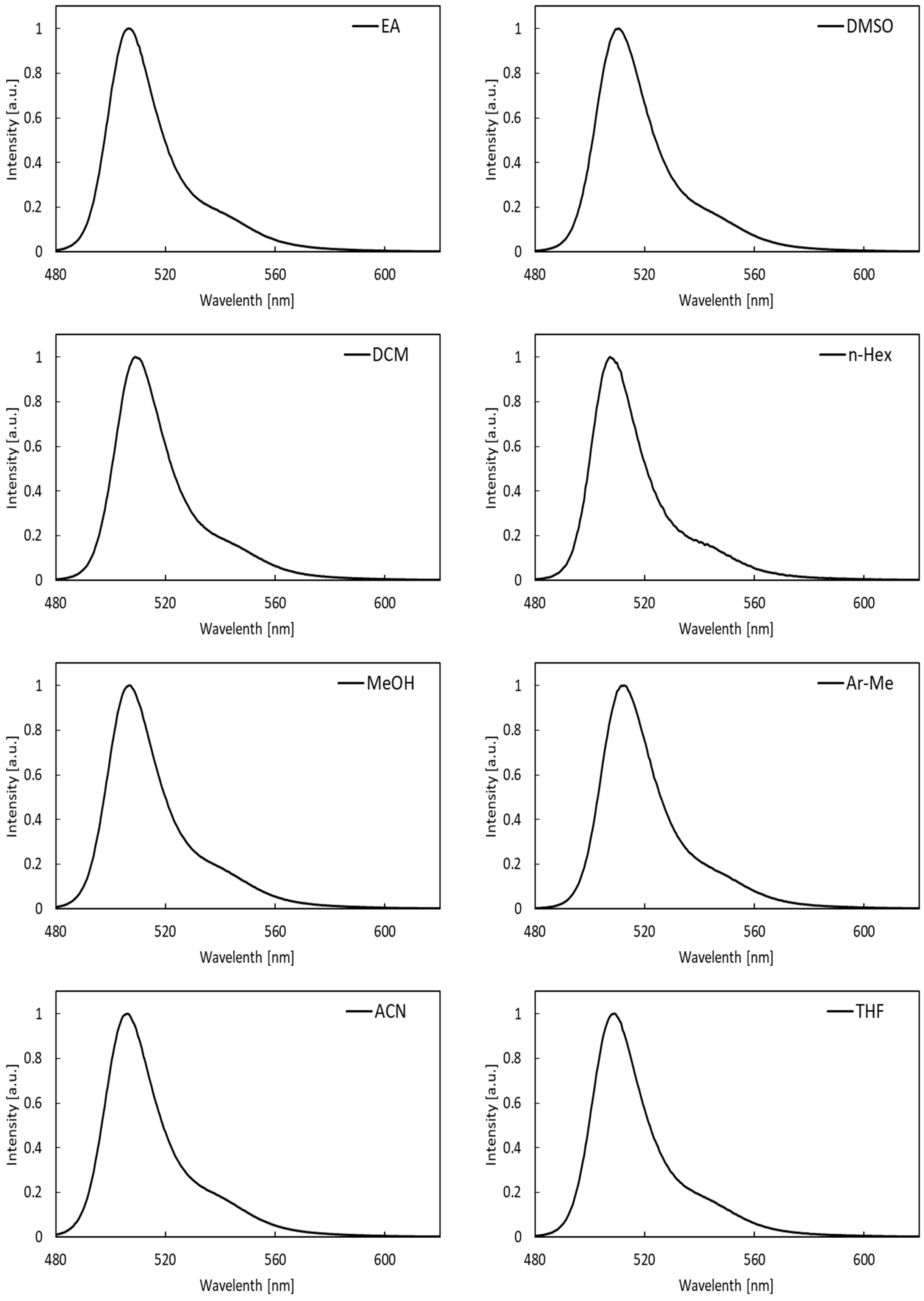

Figure S7: Fluorescence spectrum in different solvent for **1**

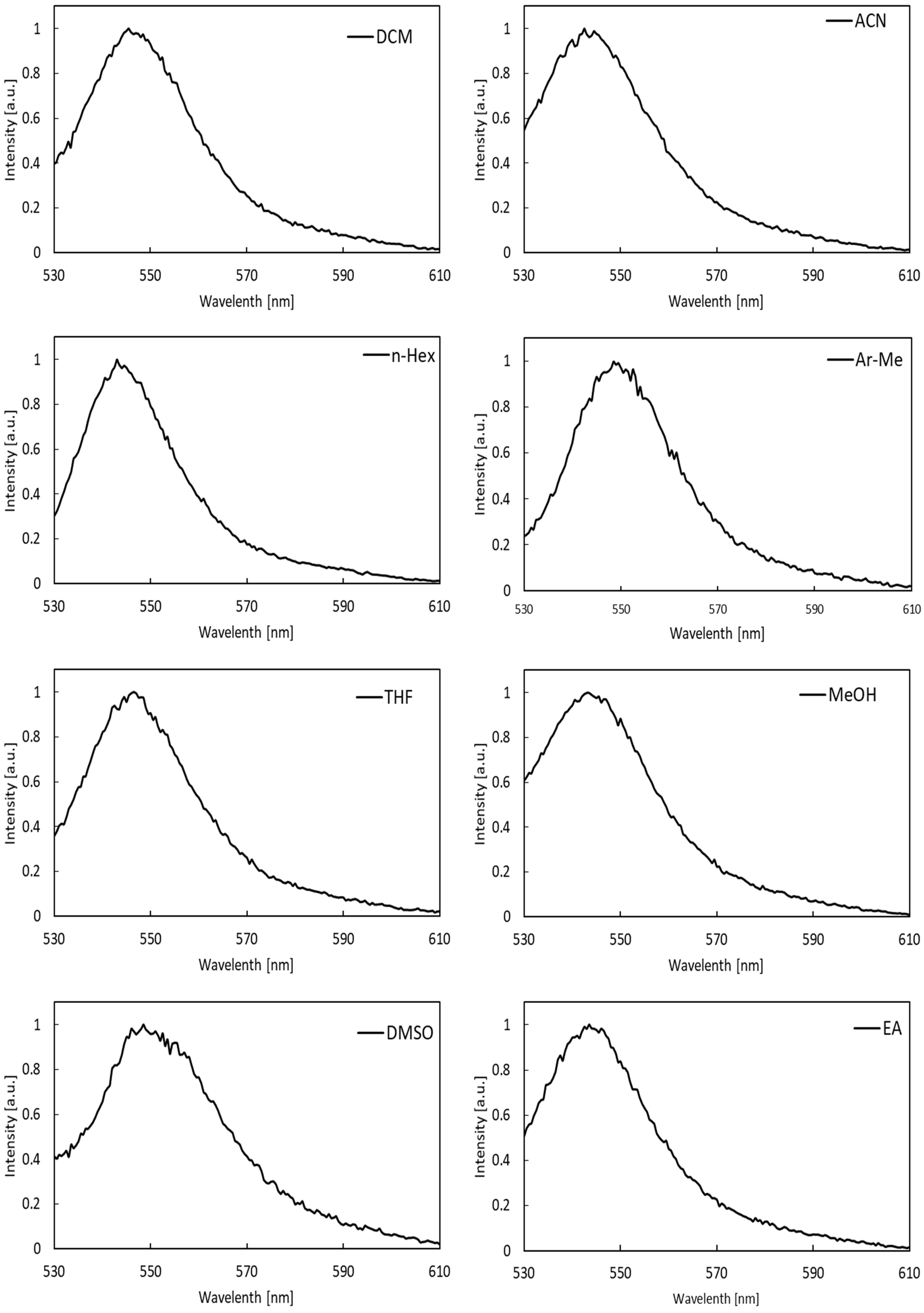

Figure S8: Fluorescence spectrum in different solvent for **2**
